# Developmental Anomalies in Human Teeth: Odontoblastic Differentiation in Hamartomatous Calcifying Hyperplastic Dental Follicles Presenting with DSP, Nestin, and HES1

**DOI:** 10.3390/jdb12010007

**Published:** 2024-01-30

**Authors:** Hiromasa Hasegawa, Katsumitsu Shimada, Takanaga Ochiai, Yasuo Okada

**Affiliations:** 1 Department of Laboratory Medicine, Shinshu University Hospital, Matsumoto 390-8621, Japan; 2 Department of Clinical Pathophysiology, Matsumoto Dental University, Shiojiri 399-0781, Japan; katsumitsu.shimada@mdu.ac.jp; 3 Department of Oral Pathology, Division of Oral Pathogenesis & Disease Control, Asahi University School of Dentistry, Mizuho 501-0296, Japan; t-ochiai@dent.asahi-u.ac.jp; 4 Department of Pathology, School of Life Dentistry at Niigata, The Nippon Dental University, Niigata 951-8580, Japan; yokada@ngt.ndu.ac.jp

**Keywords:** odontoblastic differentiation, hyperplastic dental follicle, calcifying whorled nodule, nestin, dentin sialoprotein, hairy and enhancer split 1

## Abstract

Hyperplastic dental follicles (HDFs) represent odontogenic hamartomatous lesions originating from the pericoronal tissues and are often associated with impacted or embedded teeth. These lesions may occasionally feature unique calcifying bodies, known as calcifying whorled nodules (CWNs), characterized by stromal cells arranged in a whorled or spiral fashion. CWNs are typically observed in multiple calcifying hyperplastic dental follicles or regional odontodysplasia. In our study, we examined 40 cases of HDFs, including nine instances with characteristics of CWNs, referred to as calcifying hyperplastic dental follicles (CHDFs), which are infrequently accompanied by odontodysplasia. The median ages of the HDFs and CHDFs were 16 (ranging from 3 to 66) and 15 (ranging from 11 to 50) years, respectively. The lower third molars were the most frequently affected by HDSFs and CHDFs, followed by the upper canines. A histological examination was conducted on all 40 cases, with an immunohistochemical analysis performed on 21 of them. Among the cases with CWN, nine affected a single embedded tooth, with one exception. CWNs exhibited diverse calcifications featuring sparse or entirely deposited psammoma bodies, and some displayed dentinoid formation. Immunohistochemically, the stromal cells of HDFs were frequently positive for CD56 and nestin. By contrast, CWNs were negative for CD56 but positive for nestin as well as hairy and enhancer split 1 (HES1), with a few dentin sialoprotein (DSP)-positive calcified bodies. Our results revealed that hamartomatous CHDFs can impact multiple and single-embedded teeth. CWNs composed of nestin and HES1-positive ectomesenchymal cells demonstrated the potential to differentiate into odontoblasts and contribute to dentin matrix formation under the influence of HES1. This study is the first report documenting odontoblastic differentiation in HDFs. The rare occurrence of HDFs and CHDFs contributes to limited comprehension. To prevent misdiagnosis, a better understanding of these conditions is necessary.

## 1. Introduction

Embedded or impacted teeth represent developmental anomalies that can lead to various diseases, including odontogenic cysts and tumors [1]. Hyperplastic dental follicles (HDFs) are uncommon pathological conditions associated with embedded teeth, occasionally posing a histological challenge due to their similarity to central odontogenic fibromas [2]. This lesion exhibits diverse histological features, encompassing myxofibromatous tissue, with or without various types of calcified tissue, and features reminiscent of odontogenic epithelial tumors [3,4,5]. The majority of these lesions demonstrate myxofibrous hyperplasia or a combination of odontogenic epithelial proliferation resembling ameloblastoma [5], squamous odontogenic tumors, and various calcifications, including psammoma bodies [4].

Multiple calcifying hyperplastic dental follicles (MCHDFs) represent a rare pathological condition that was initially described by Sandler et al. in 1988 [6]. The term MCHDF was coined by Gardner DG and Radden B in 1995 [7], and subsequently, numerous authors reported similar cases using this nomenclature [8,9,10,11,12,13,14]. These cases share common histological features, displaying small droplets of calcified material with a whorled pattern and varying amounts of odontogenic epithelial islands within fibrous or myxofibrous tissue. Furthermore, some instances are accompanied by regional odontodysplasia [7]. This type of calcification can be found in solitary hyperplastic dental follicles, occasionally forming conglomerates of enameloid, cementoid, and psammomatous calcifications. The initiation of tooth formation involves the following two integral components: the surface epithelium and the underlying mesenchyme, the latter originating from cranial neural crest cells [15]. Notably, human dental pulp stem cells exhibit positivity for CD24, CD117, and CD146 while lacking nestin expression [16,17]. In contrast, dental follicle spindle cells, characterized by a notable population of neural stem/progenitor cells expressing nestin [18], demonstrate the capability to differentiate into neural cells [19]. Both immature and mature odontoblasts, derived from neural crest cells, express nestin, the dentin sialoprotein (DSP), and mRNA [20,21].

This study focused on the examination of the structure of small droplets of calcified material exhibiting a distinctive whorled or spiral pattern, termed calcifying whorled nodules (CWNs). While CWN is a characteristic feature of MCHDF, it is also identified in isolated cases of calcifying hyperplastic dental follicles (CHDFs). The hypothesis posits that solitary or multiple CHDF instances may constitute the hamartomatous overgrowth of neural crest cells that incompletely differentiate into odontoblasts, resulting in the formation of dentinoids or calcified particles. To elucidate the nature of CWNs, we conducted immunohistochemical investigations on dental follicles, including CHDFs, carefully selected from our archival specimens. This presentation provides compelling immunohistochemical profiles of CWNs, shedding light on the potential odontoblastic differentiation within dental follicles.

## 2. Materials and Methods

### 2.1. Sample Characteristics

Formalin-fixed, ethylenediaminetetraacetic acid-treated, and paraffin-embedded (FFPE) human samples were sourced from the archives of the Department of Surgical Pathology at Matsumoto Dental University Hospital. The inclusion criteria were as follows: (1) radiolucent lesions around the embedded tooth/teeth; (2) fibrous or myxoid stroma with or without small dentinoids; and (3) markedly thickened cyst walls composed of significant myxoid stroma or dentinoid hard tissue in odontogenic cysts such as dentigerous cysts or odontogenic keratocysts caused by hyperplastic dental follicles. Patients with odontomas and dominant hard tissue formation composed of dentinoid and enameloid tissues were excluded. In total, 40 samples were obtained from our archives, with the anterior incisors, premolars, and molars accounting for 30%, 5%, and 65% of the 40 patients, respectively.

Third molars with incomplete root formation, extracted under the diagnosis of unerupted teeth, served as positive control tissues for immunohistochemical analyses. This study adhered to the principles of the 2008 Declaration of Helsinki, and the study protocol received approval from the Ethics Committee of Matsumoto Dental University (approval number 0322).

### 2.2. Immunohistochemistry

Histological sections, three micrometers thick, were prepared from FFPE samples. Following deparaffinization and hydration, optimized antigen-retrieval methods were employed, as outlined in Table 1 (dentin sialoprotein (DSP): proteinase K for 3 min; CD56/NCAM and CD117/c-kit: heat-induced epitope retrieval (HIER) in a pressure cooker with antigen-retrieval solution (pH 6.0) (#715281, Nichirei Bioscience, Tokyo, Japan); hairy and enhancer split 1 (HES1); and nestin: HIER in a pressure cooker with antigen-retrieval solution (pH 9.0) (#415201, Nichirei Bioscience)). Endogenous peroxidase activity was blocked due to incubation in a 3% H_2_O_2_ aqueous solution for 15 min, and nonspecific reactions were blocked due to incubation in a protein block solution (#X090930-2, Agilent Technologies, Santa Clara, CA, USA) for 10 min. After incubation with primary antibodies for 1 h at 24 °C, the sections were washed three times with phosphate-buffered saline and incubated with a horseradish peroxidase-conjugated secondary antibody (#424152, Histofine Simple Stain MAX PO Multi, Nichirei Bioscience) for 30 min. For visualization, sections were incubated with 3-3′-diaminobenzidine tetrahydrochloride (DAB) (#K3468, Agilent Technologies), then counterstained with hematoxylin.

Third molar sections, displaying incomplete root formation, were employed as positive controls for the immunohistochemical analyses. Negative control sections were meticulously prepared using this procedure, except for omitting the primary antibodies. This approach aimed to discern and identify any nonspecific reactions.

### 2.3. Statistical Analysis

The age distributions of patients, both with and without CWN, were subjected to analysis through the Mann–Whitney U test. An examination of the locations of CHDFs, nestin, HES1, and DSP between CHDFs and other HDFs, as well as the comparison of HES1 immunoreactions between DSP-positive and negative cases, was conducted utilizing Fisher’s exact test. Post hoc analysis was performed using Holm’s test. All statistical analyses were performed using EZR (Saitama Medical Center, Jichi Medical University, Saitama, Japan) [22]. EZR is a user-friendly graphical interface for R www.r-project.org (R Foundation for Statistical Computing, Vienna, Austria). *p*-values < 0.05 were considered statistically significant.

## 3. Results

### 3.1. Clinical Characteristics of CHDFs

**Figure 1:**
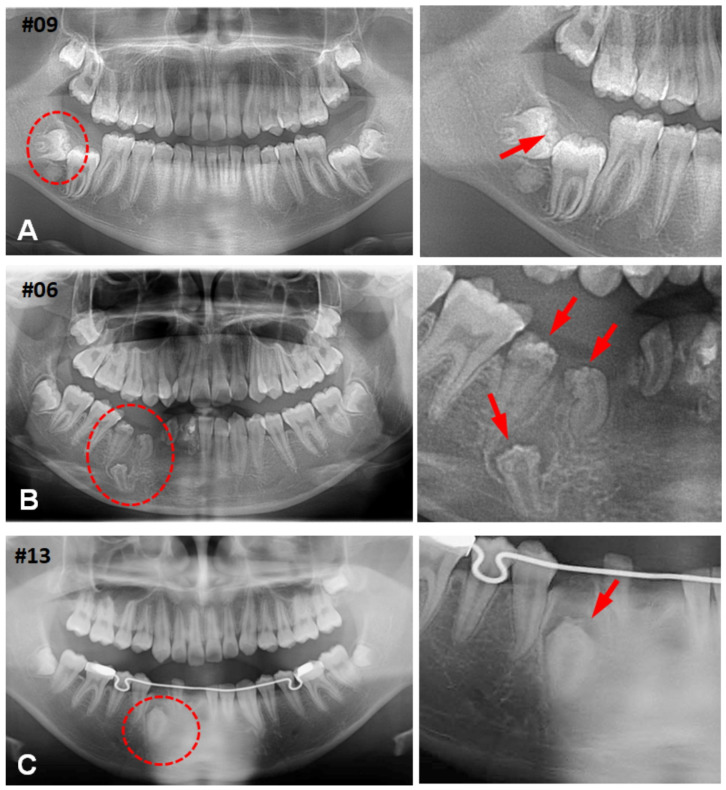
Representative dental panoramic radiographic images and enlarged pictures of dotted circles: a hyperplastic dental follicle (arrow) in case 9 affecting the impacted mandibular third molar (**A**); multiple calcifying hyperplastic dental follicles (arrows) in case 6 (**B**); and a solitary hyperplastic dental follicle (arrow) in case 13 (**C**).

In this study, we examined 40 cases of HDFs to explore the clinical characteristics of CHDFs. Notably, nine cases (22%) exhibited CWNs as a distinctive feature of MCHDFs. Only two cases (5%) displayed odontodysplasia, accompanied by MCHDF and solitary CHDF. The age distributions of HDFs and CHDFs ranged from 3 to 66 years and 11 to 50 years, respectively. Statistical analysis revealed no significant difference (*p* = 0.228) in the median ages of all HDFs and CHDFs, which were 16 and 15 years, respectively. Sex predilection was not observed. The molar region was the most frequently affected by both HDFs (67.7%) and CHDFs (55.6%), followed by the anterior teeth (29%, 33.3%) and premolars (3.2%, 11.1%), with no statistically significant difference (*p* = 0.426). CHDFs predominantly developed in the lower third molars (44.4%), followed by the upper anterior teeth (33.3%) (Figure 1, Table S1). Multiple teeth were affected, including the lower canines and premolars, in one case (Figure 1B).

The initial clinical diagnoses of all 40 HDFs included variable dentigerous cysts, embedded or impacted teeth, odontogenic tumors, odontogenic keratocysts, ameloblastomas, odontogenic fibromas, and mandibular cysts. Dentigerous cysts were most frequently suspected, accounting for 20 cases (50%), followed by 10 cases (25%) of odontogenic tumors, 4 cases (10%) of embedded or impacted teeth, and 3 cases (8%) of odontogenic keratocyst, among others. Similarly, of the 9 CHDFs, dentigerous cysts and odontogenic tumors accounted for 6 (67%), 2 (22%), and 1 (11%) (Table S1).

### 3.2. Histological Changes of HDFs

**Figure 2:**
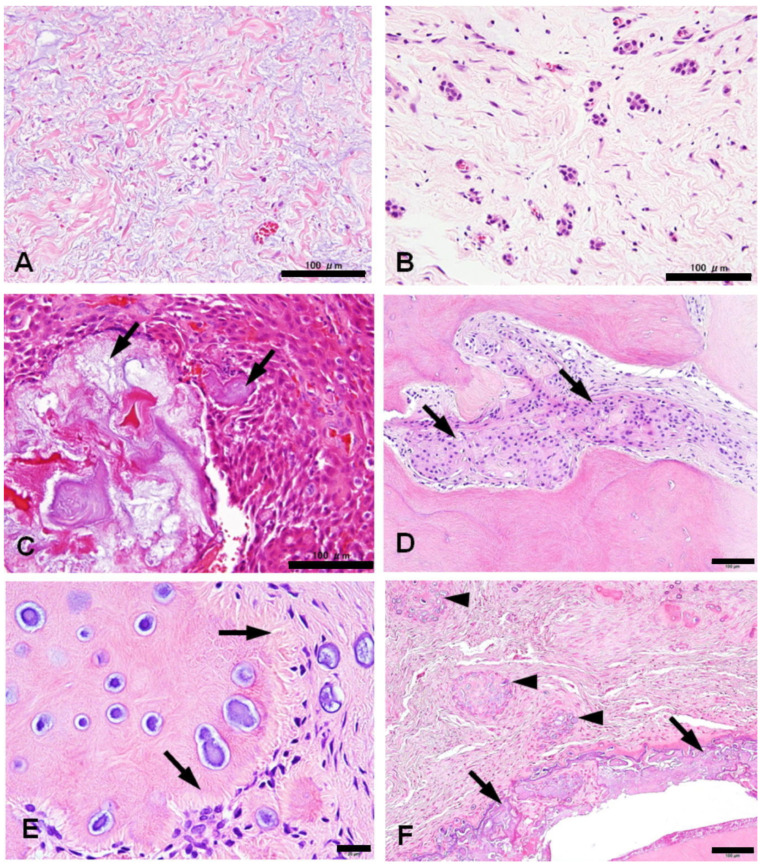
Histological variations in hyperplastic dental follicles: the stroma of the lesion is frequently myxo-fibrous (**A**), accompanied by the proliferation of small islands of the odontogenic epithelium (**B**); rarely, enamloid materials are present in the proliferated epithelium (**C**); dentinoid formation frequently accompanies juxtaposed odontogenic epithelium (arrows) (**D**); some cases show dentinoid nodules with a structure reminiscent of dentinal tubules (arrows) and psammomatous calcification surrounded by spindle cells forming a whorled structure, namely a calcifying whorled nodule (**E**); the representative case 13 shows many calcifying whorled nodules (arrowheads) distributed around a tooth crown with odontodysplasia (arrows) (**F**). All the bars represent 100 µm except for (**E**) (20 µm).

The fibrous lesions that enveloped the crowns of the impacted teeth exhibited varying degrees of thickening, often displaying fibrous changes in 79% of cases and myxoid changes in 67%. Giant cells were detected in only one instance, comprising 3% of the cases. Most cases (95%) featured diverse numbers of epithelial islands. In 36% of cases, the reduced enamel epithelium exhibited reticular or massive proliferation, occasionally accompanied by ghost cells (18%) or the deposition of enamloid matrices (5%). Mesenchymal calcification was observed in approximately half of the cases, categorized into the following three types: psammomatoid calcification (49%), calcifying whorled nodules (23%), and dentinoid formation (41%). The dentinoid hard tissues had irregular tubular structures with no cells embedded in the matrix throughout their bodies (Figure 2 and Table S2).

**Figure 3:**
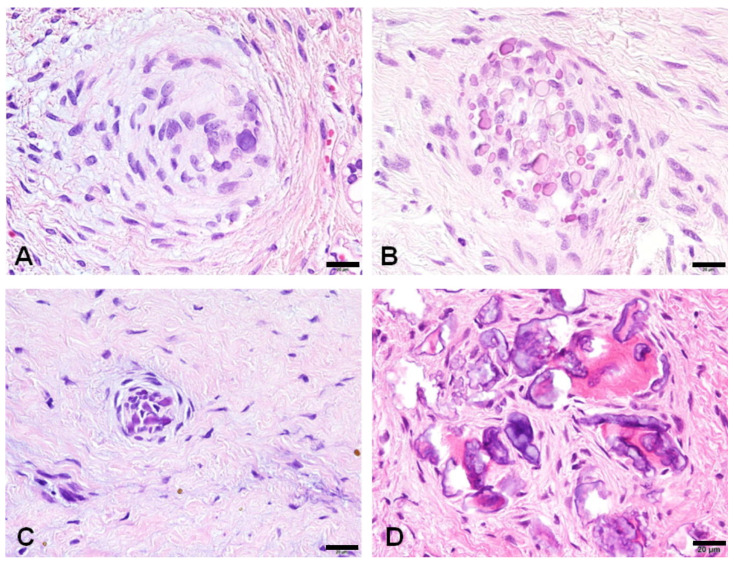
Histological variations of calcifying whorled nodules: spindle cells aggregate in a whorled or spiral fashion with scant calcification (**A**); some nodules show an increased deposition of small calcification (**B**); occasionally, a small whorled nodule is occupied by psammoma bodies (**C**); some nodules develop dentinoid formation around the psammoma bodies (**D**). All the bars represent 20 µm.

Calcifying whorled nodules exhibited diverse forms of calcification. Certain nodules consisted of spindle cells gathering in a spiral configuration with minimal calcification. Other nodules demonstrated the heightened deposition of small calcifications. On occasion, a diminutive whorled nodule contained psammoma bodies, and certain nodules underwent dentinoid formation around the psammoma bodies (Figure 3).

### 3.3. Immunohistochemical Profiles of CWNs in CHDFs

**Figure 4:**
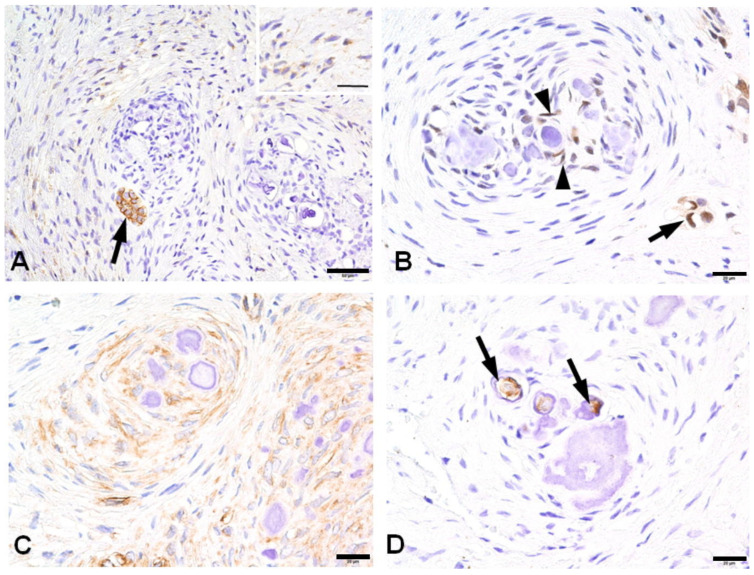
The immunohistochemical findings of calcifying whorled nodules: stromal spindle cells are positive for CD56 (inset), but calcifying whorled nodules are negative. There is a CD56-positive epithelial island (arrow) (**A**); a few spindle cells (arrowheads) in a calcifying whorled nodule and some epithelial cells (arrows) are nuclear-positive for hairy and enhancer split-1 (HES1) (**B**); spindle cells aggregating in a wheel fashion are entirely positive for nestin (**C**); the central areas of concentric calcified materials (arrow) are focally positive for dentin sialoprotein (DSP) (**D**). All the bars represent 20 µm except for (**A**) (50 µm).

Immunohistochemically, the stromal spindle cells of CHDFs exhibited positive CD56 expression in most cases (81.0%), while the spindle cells of CWNs were consistently negative. Focal CD56-positive epithelial islands were identified in 66.7% of cases. HES1 demonstrated rare positivity in stromal spindle cells (6.7%), contrasting with the positive expression observed in 66.7% of spindle cells in CWNs and 56.3% of odontogenic epithelium cases. Nestin displayed positive expression in 95% of stromal spindle cells and 100% of CWNs, yet it was completely negative in the odontogenic epithelium. The central areas of concentric calcified material in CWNs were only focally positive for DSP in 50% of cases. Notably, all DSP-positive cases exhibited positivity for nestin, and 75% of DSP-positive cases were positive for HES1 (Tables S2 and S3). Ultimately, this study elucidated a propensity for positive reactions to nestin, HES1, and DSP in the spindle cells of CWNs. CD117 exhibited complete negativity in all cases (Figure 4 and Table 2). The immunoreactivity of stromal spindle cells against CD56 and nestin demonstrated no significant difference between CHDFs and non-CHDF/HDFs without CWNs (CD56, *p* = 0.618; nestin, *p* = 1).

## 4. Discussion

Aberrant psammomatous calcification may manifest in the syndromic or regional hypoplasia of teeth, albeit infrequently impacting dentition. Regional odontodysplasia, initially documented by Hitchin in 1934, exhibits cementum-like and psammomatous calcification, enveloped by a whorled fibrous stroma referred to as CWN in this investigation [23]. Multiple calcifying hyperplastic dental follicles represent a rare condition characterized by numerous unerupted teeth with substantial calcifications and remnants of odontogenic epithelium within their enlarged dental follicles, often accompanied by odontodysplasia [6,7,8,9,10,11,14]. Histologically, all cases demonstrated similarities, showcasing calcified materials with a whorled or spiral structure akin to CWN. HDFs are odontogenic hamartomatous lesions arising from pericoronal tissues associated with impacted or embedded teeth [24], presenting diverse histological findings, as previously documented [4]. While CWNs are traditionally considered a distinctive feature of MCHDFs, this study reveals that CWNs may also manifest in solitary embedded teeth exhibiting hyperplastic changes, denoted as solitary CHDFs.

Notably, this study breaks new ground by providing immunohistochemical insights into the stromal cells of CWNs, which is a facet not previously explored. The immunohistochemical profile of CWNs appears to align with that of odontoblasts during the latter stages of differentiation.

The dental follicles of impacted third molars infrequently contain immature fibroblasts exhibiting the phenotypic features of stromal stem cells expressing CD44 and CD56, constituting less than 2% [25]. In contrast, CD56-positive cells were identified in 81% of the cases within our series. This observation underscores the prevalent presence of stromal stem cells in HDFs, suggesting their capacity for differentiation into odontoblasts. Notably, HDFs diverge from dental pulp stem cells, as the latter express CD117/c-kit [17], a marker that was absent in all HDF cases analyzed. This finding elucidates the absence of odontogenic components associated with odontomas in HDFs. Morphological examinations further support this conclusion, as no calcified tissue consisting of dentinoid and enameled tissues was observed in HDFs. This precludes the possibility of minute odontomas originating from impacted teeth.

To comprehend tooth initiation, it is imperative to consider the following two fundamental components: the surface epithelium and the underlying mesenchyme. The dental mesenchyme, crucial for tooth development, originates from cranial neural crest cells migrating into the frontonasal process and the first branchial arch [15]. Nestin, initially characterized by neural stem cells, is a cytoskeletal intermediate filament and serves as a neuroepithelial stem cell marker. Despite its association with neural cells, recent evidence from in vivo models and humans highlights the presence of nestin-positive cells with progenitor and/or regulatory functions in various tissues [26]. Notably, odontogenic ectomesenchymal cells, akin to neural cells in origin [15], exhibit convincing evidence of nestin. The dental follicle, an ectomesenchymal tissue that envelops the developing tooth germ, is a noteworthy example. Human follicle cells distinctively demonstrate the ability to differentiate into neural cells. In cultured spindling dental follicle cells, the expression of neural markers such as nestin, β-III-tubulin, and S100β is evident. Additionally, there is an upregulation of Musashi-1 and Musashi-2, MAP2, GFAP, MBP, and SOX10 [19].

Nestin serves as a prominent dentin follicle marker [27] and is notably expressed in differentiated odontoblasts [21]. While there is limited documentation on nestin expression in odontogenic neoplasms or related lesions, it has been observed that all ameloblastomas and malignant ameloblastomas exhibit negativity for nestin. However, in mixed odontogenic tumors, nestin immunoreactivity is evident in the odontogenic ectomesenchyme, particularly in regions adjacent to the odontogenic epithelium. Moreover, positive nestin expression is observed in odontoblasts, pulp cells, and cells adhering to dentin in odontomas [28]. Nestin has also been utilized as a differentiation marker for odontoblasts during tooth development. In the later stages of odontogenesis, the unmineralized matrix gradually transforms into mature dentin. In this context, DSP and nestin are recognized as markers of late-stage odontoblastic differentiation [29]. Notably, experimental evidence indicates the co-expression of nestin and DSP during the late stage of odontoblast differentiation, encompassing both pre-odontoblasts and mature odontoblasts [20]. The DSP domain plays a crucial role in facilitating mesenchymal cell differentiation and mineralization [30]. Calcifying whorled structures exhibit positive reactions to nestin and DSP, which is indicative of the late stage of odontoblast differentiation. Morphologically, some calcifying whorled structures contain dentinoid materials with tubule-like structures reminiscent of dentinal tubules. This structure provides simple and reliable evidence showing the presence of mesenchymal cells that differentiate into odontoblasts in dental follicles. Considering both the immunohistochemical findings and the morphological features of dentinoid formation, it is conceivable that certain constituent cells of dental follicles possess the potential to undergo differentiation into odontoblasts. This report represents the initial documentation of odontoblastic differentiation in HDFs.

The Notch signaling pathway holds paramount status as an ancient and remarkably preserved mechanism. Its involvement extends across diverse physiological processes, including cell differentiation and regeneration, while also playing a pivotal role in pathological conditions such as carcinogenesis. Specifically, the signaling of Notch operates in a manner that negatively regulates the differentiation of neural stem cells, directing them away from the neuronal lineage [31]. Genes featuring suppressors of hairless paired sites (SPS) or Notch transcription complexes at DNA-binding sites are inclined to be high-affinity targets for Notch. Consequently, Hes1, a prototypical SPS-containing Notch target, exhibits responsiveness to Notch1 [32]. The upregulation of Hes1 may be attributed to increased activity within the Notch signaling pathway, potentially contributing to the proliferation of tumors [33]. Conversely, Hes1 acts as an inhibitor of Notch signaling, thereby influencing the differentiation of embryonic stem cells through the suppression of Notch signaling via Hes1 [34]. The expression of Hes1 has been identified in the stellate reticulum cells and the outer dental mesenchyme [35]. In dental pulp subjected to Ca(OH)_2_ treatment, there was an observed upregulation of Hes1, accompanied by the increased expression of Notch1, Notch2, and Notch3. Conversely, diminished Notch/Hes1 signaling has been linked to a reduction in tooth size [36]. These investigations indicate that Hes1 plays a significant role in odontogenesis. Furthermore, the suppression of Notch signaling has been found to facilitate odontoblastic differentiation in HES1-positive CWN cells.

In vivo, hyperplastic dental follicles potentially serve as valuable models for understanding odontogenesis in human ectomesenchymal cells. Unfortunately, the scope of this study was limited due to the availability of specimens suitable for immunohistochemical examination, and none were deemed appropriate for molecular analysis. Consequently, our findings indicate a tendency for the immunophenotypes of constituent cells within CWNs to express nestin, DSP, and HES1. However, it is imperative to underscore that additional investigations are imperative to substantiate and validate these preliminary results.

HDFs or CHDFs frequently occur in the mandibular molars, followed by the maxillary anterior teeth. This tendency is compatible with the frequency of impacted teeth [37]. Barring multiple CHDFs, the discrimination between single CHDFs and odontogenic cysts/tumors is difficult because these diseases share similar clinical characteristics. As another limitation, since we focused on histological findings, especially the immune phenotype of proliferating mesenchymal cells, our study lacked detailed examinations of radiographic or other clinical characteristics.

Our case series found that the majority of HDFs were diagnosed with odontogenic cysts or odontogenic tumors. CHDFs had a similar tendency. In particular, half of HDFs and over half of CHDFs were clinically diagnosed with a dentigerous cyst. Previous reports also showed that most cases of clinical diagnosis were dentigerous cysts [3]. As shown in representative dental panoramic radiographic images, it is reasonable that the radiolucent area around embedded tooth crowns, which expands at variable degrees, gives us an impression of a cystic lesion. Dentinoid formation could be found in 41% of HDGS; the cases with radiopaque findings might be misinterpreted as some odontogenic tumors. It appears that these conditions are not widely known yet due to the rarity of HDFS and CHDFs. Pathologists may face diagnostic confusion due to the initial clinical diagnosis of most cases as odontogenic cysts or odontogenic tumors. Therefore, further investigations are necessary to clarify not only the biological basis of HDFs and HDFs but also the clinical characteristics of these conditions to make accurate clinical diagnoses.

## 5. Conclusions

Hamartomatous calcifying hyperplastic dental follicles not only impact multiple teeth but also manifest in single embedded teeth. Remarkably, characteristic features of MCHDs, such as CWNs, may also be present in solitary HDFs without concurrent odontodysplasia. The transition from a CD56-positive to a CD56-negative status in ectomesenchymal cells of HDFs is noteworthy. Subsequently, mesenchymal cells forming whorled nodules may harbor the potential to differentiate into odontoblasts, thereby producing a dentin matrix under the influential guidance of HES1. The clinical and pathological conditions of HDFs or CHDFs should be understood to avoid the misinterpretation or misdiagnosis of these lesions.

## Figures and Tables

**Table 1:** Panel of primary antibodies.

Antibody	Product Number	Source	Clone	Treatment	Dilution
CD56/NCAM	M730429-2	Agilent Technologies, Santa Clara, CA, USA	123C3	HIER	1:100
CD117/c-kit	A450229-2	Agilent Technologies, Santa Clara, CA, USA	polyclonal	HIER	1:50
DSP	sc-73632	Santa Cruz Biotechnology Inc., Dallas, TX, USA	polyclonal	Digestion	1:4000
HES1	#11988	Cell Signaling Technology Inc., Danvers, MA, USA	D6P2U	HIER *	1:200
Nestin	HPA007007	Sigma-Aldrich, St. Louis, MO, USA	polyclonal	HIER *	1:5000

DSP: dentin sialoprotein; HES1: hairy and enhancer split 1; HIER: heat-induced epitope retrieval (pH 6.0), * (pH 9.0).

**Table 2:** Summary of immunophenotypes of components of hyperplastic dental follicles.

Components	CD56	Nestin	HES1	CD117	DSP
Stroma	+	+	+	−	−
Epithelial islands	+	−	+	−	−
CWNs	−	+	+	−	+
Psammoma bodies	−	−	−	−	−
Dentinoid	−	−	−	−	−

CWNs: calcifying whorled nodules; HES1: hairy and enhancer split 1; DSP: dentin sialoprotein; +: positive; −: negative.

## Data Availability

All data were included in this manuscript and three Supplemental Tables.

## Supplementary Materials

The following supporting information can be downloaded at: https://www.mdpi.com/article/10.3390/jdb12010007/s1, Table S1: Clinical summary of HDFs; Table S2: Histological findings of HDFs; Table S3: Immunohistochemical findings of HDFs.
