# Supplementary material for: Developmental Anomalies in Human Teeth: Odontoblastic Differentiation in Hamartomatous Calcifying Hyperplastic Dental Follicles Presenting with DSP, Nestin, and HES1
Source: J Dev Biol. 2024 Jan 30;12(1):7. doi: 10.3390/jdb12010007 (PMC10885117; doi:10.3390/jdb12010007)
Supplement: Supplementary file 1 [file jdb-12-00007-s001.zip › Table S1 Clinical summary of HDFs.pdf]

Table S1. Clinical Summary

Case	Age	Sex	Location	Clinical diagnosis
HDF1	11	F	38	ameloblastoma
HDF2	13	F	13	odontogenic fibroma
HDF3	11	F	21	embedded tooth
HDF4	37	F	48	odontogenic tumor
HDF5	14	F	37	embedded tooth
HDF6	15	F	43,44,45	odontogenic keratocyst
HDF7	14	F	45	odontogenic tumor
HDF8	66	F	38	dentigerous cyst
HDF9	17	M	48	dentigerous cyst
HDF10	12	F	47	dentigerous cyst
HDF11	27	F	13,23	dentigerous cyst
HDF12	49	F	48	odontogenic tumor
HDF13	15	M	13	dentigerous cyst
HDF14	26	M	28	odontogenic tumor
HDF15	50	M	38	dentigerous cyst
HDF16	56	M	18	dentigerous cyst
HDF17	15	M	48	odontogenic keratocyst
HDF18	20	F	38	odontogenic tumor
HDF19	11	F	13,23	odontogenic tumor
HDF20	12	M	13	dentigerous cyst
HDF21	15	F	36	dentigerous cyst
HDF22	16	F	42	odontogenic tumor
HDF23	16	F	42	odontogenic tumor
HDF24	16	M	38	dentigerous cyst
HDF25	12	F	47	dentigerous cyst
HDF26	15	M	37	dentigerous cyst
HDF27	11	M	13	dentigerous cyst
HDF28	6	M	11	odontogenic tumor
HDF29	13	F	21	odontogenic tumor
HDF30	48	F	38	dentigerous cyst
HDF31	43	M	38	odontogenic keratocyst
HDF32	13	M	16	dentigerous cyst
HDF33	15	M	48	dentigerous cyst
HDF34	15	M	38	dentigerous cyst
HDF35	23	M	47	dentigerous cyst
HDF36	22	F	48	dentigerous cyst
HDF37	16	M	38	mandibular cyst
HDF38	50	F	48	dentigerous cyst
HDF39	15	M	38	Impacted tooth
HDF40	3	F	51	Impacted tooth

HDF: hyperplastic dental follicle; Location: described by the FDI World Dental Federation numbering system
