# Supplementary material for: Developmental Anomalies in Human Teeth: Odontoblastic Differentiation in Hamartomatous Calcifying Hyperplastic Dental Follicles Presenting with DSP, Nestin, and HES1
Source: J Dev Biol. 2024 Jan 30;12(1):7. doi: 10.3390/jdb12010007 (PMC10885117; doi:10.3390/jdb12010007)
Supplement: Supplementary file 1 [file jdb-12-00007-s001.zip › Table S2 Histological findings of HDFs .pdf]

Table S2. Histological findings of hyperplastic dental folliciles (HDFs)

Case	Cyst formation		Odonto-dysplasia	Stroma			Epithelium				Hard tissue formation			
		(type)		Fibrous	Myxoid	Giant cell	Epithelial island	Epithelial proliferation	(type)	Ghost cell	Enameloid	Psammo-matous	Dentinoid	Calcifying whorled nodule
HDF1	-		-	+	-	-	+	-		-	-	-	-	-
HDF2	-		-	+	-	-	+	-		-	-	-	-	-
HDF3	-		-	-	-	-	+	+	DGCT	+	+	-	+	-
HDF4	-		-	-	+	-	+	-		-	-	-	+	-
HDF5	-		-	+	+	-	+	-		-	-	-	+	-
HDF6	-		+	+	+	-	+	-		-	-	+	+	+
HDF7	-		-	+	+	-	+	+	DGCT	-	-	-	+	-
HDF8	+	DC	-	+	-	-	+	+	DGCT	-	-	-	+	-
HDF9	-		-	+	+	-	+	-		-	-	+	+	+
HDF10	-		-	+	+	-	+	-		+	-	-	+	-
HDF11	-		-	+	+	-	+	-		-	-	-	+	-
HDF12	-		-	+	-	-	+	-		-	-	+	+	+
HDF13	-		+	+	+	-	+	-		-	-	+	+	+
HDF14	+	DC	-	+	-	-	+	-		-	-	+	+	+
HDF15	+	DC	-	+	-	-	-	-		-	-	+	+	+
HDF16	+	DC	-	+	-	-	-	-		-	-	+	+	-
HDF17	+	OKC	-	-	+	-	+	-		-	-	-	-	-
HDF18	+	OKC	-	-	+	-	+	-		-	-	-	-	-
HDF19	-		-	+	+	-	+	-		+	-	+	-	-
HDF20	-		-	+	-	-	+	-		-	-	-	-	-
HDF21	-		-	+	+	-	+	+		+	+	-	+	-
HDF22	-		-	+	+	-	+	+		-	-	+	-	+
HDF23	-		-	+	+	-	+	+		-	-	+	-	-
HDF24	-		-	+	+	-	+	-		-	-	+	-	+
HDF25	-		-	+	+	+	+	-		-	-	-	-	-
HDF26	-		-	+	+	-	+	+	DGCT	+	+	+	+	-
HDF27	-		-	-	+	-	+	+		-	-	+	-	+
HDF28	-		-	-	+	-	+	-		-	-	-	-	-
HDF29	-		-	+	-	-	+	+	AOT	-	-	+	-	-
HDF30	+	DC	-	-	-	-	+	+		-	-	-	-	-
HDF31	+	OKC	-	-	+	-	+	+		-	-	-	-	-
HDF32	-		-	+	+	-	+	-		-	-	-	-	-
HDF33	-		-	+	+	-	+	+	DGCT	+	-	+	+	-
HDF34	-		-	+	+	-	+	-		-	-	-	-	-
HDF35	-		-	+	+	-	+	-		-	-	-	-	-
HDF36	-		-	+	+	-	+	-		-	-	-	-	-
HDF37	+	OKC	-	+	+	-	+	-		-	-	-	-	-
HDF38	+	DC	-	+	-	-	+	+		-	-	-	+	-
HDF39	-		-	+	+	-	+	+	DGCT	+	-	+	+	-
HDF40					+	-	+	+		-	-	-	-	-

DC: dentigerous cyst; OKC: odontogenic keratocyst; DGCT: dentinogenic ghost cell tumor-like; AOT: adenomatoid odontogenic tumor-like
