# Supplementary material for: Developmental Anomalies in Human Teeth: Odontoblastic Differentiation in Hamartomatous Calcifying Hyperplastic Dental Follicles Presenting with DSP, Nestin, and HES1
Source: J Dev Biol. 2024 Jan 30;12(1):7. doi: 10.3390/jdb12010007 (PMC10885117; doi:10.3390/jdb12010007)
Supplement: Supplementary file 1 [file jdb-12-00007-s001.zip › Table S3 Immunohistochemical findings of HDFs .pdf]

Table S3. Immunohistochemical results of hyperplastic dental follicles (HDFs)

Case	CD56 (E/S/W)	nestin (E/S/W)	HES1 (E/S/W)	CD117 (E/S/W)	DSP
HDF1					
HDF2					
HDF3					
HDF4					
HDF5					
HDF6	+/+/-	-/+/+	+/-/+	-/-/-	+
HDF7	-/+/na	-/+/na	+/-/na		-
HDF8	+/+/na	-/+/na	-/-/na		-
HDF9	+/+/-	-/+/+	+/-/+	-/-/-	-
HDF10	+/-/na	-/+/na	-/-/na		-
HDF11					
HDF12	+/+/-	-/-/+			+
HDF13	+/+/-	-/+/+	+/-/+	-/-/-	+
HDF14	-/-/na	-/+/na	-/-/na		-
HDF15	+/-/-	-/+/+	-/-/-		-
HDF16	na/-/na	na-+/na			
HDF17	+/+/na	-/+/na			
HDF18	+/+/na	-/+/na			
HDF19	-/+/na	-/+/na	-/-/na		-
HDF20	+/+/na	-/+/na	+/-/na		-
HDF21	+/+/na	-/+/na	-/-/na		
HDF22	+/+/-	-/+/+	+/-/+	-/-/-	+
HDF23	-/+/na	-/+/na	+/-/na		-
HDF24	-/+/na	-/+/+	-/-/-		-
HDF25	-/+/na	-/+/na	+/-/na		
HDF26					
HDF27					
HDF28	na/+/na	na/+/na	na/-/na		
HDF29					
HDF30					
HDF31					
HDF32					
HDF33					
HDF34					
HDF35					
HDF36					
HDF37					
HDF38					
HDF39	-/-/na	-/-/na	+/-/na		+
HDF40					

E: epithelium; S: stroma; W: calcifying whorled nodule; na: not analyzed due to the absence of objects; Blank column: specimen not available for staining
